# WNT ligands in non-small cell lung cancer: from pathogenesis to clinical practice

**DOI:** 10.1007/s12672-023-00739-7

**Published:** 2023-07-24

**Authors:** Wanting Xue, Lihong Cai, Su Li, Yujia Hou, Yan-Dong Wang, Dongbin Yang, Yubing Xia, Xiaobo Nie

**Affiliations:** 1 Key Laboratory of Receptors-Mediated Gene Regulation and Drug Discovery, School of Basic Medical Sciences, Hebi Key Laboratory of Liver Disease, People’s Hospital of Hebi, Henan University, Kaifeng, Hebi, China; 2 Kaifeng Key Laboratory of Radiation Oncology, Kaifeng Cancer Hospital, Kaifeng University, Kaifeng, 475003 China; 3 State Key Laboratory of Chemical Resource Engineering, College of Life Science and Technology, Beijing University of Chemical Technology, Beijing, China; 4 School of Basic Medical Sciences, Henan University, Kaifeng, 475004 China; 5 Hebi Key Laboratory of Liver Disease, People’s Hospital of Hebi, Henan University, Hebi, 458030 China

**Keywords:** WNT ligands, Non-small cell lung cancer, WNT signaling pathways, Therapeutic targets

## Abstract

Non-small cell lung cancer (NSCLC) is the malignant tumor with the highest morbidity and leading cause of death worldwide, whereas its pathogenesis has not been fully elucidated. Although mutations in some crucial genes in WNT pathways such as β-catenin and APC are not common in NSCLC, the abnormal signal transduction of WNT pathways is still closely related to the occurrence and progression of NSCLC. WNT ligands (WNTs) are a class of secreted glycoproteins that activate WNT pathways through binding to their receptors and play important regulatory roles in embryonic development, cell differentiation, and tissue regeneration. Therefore, the abnormal expression or dysfunction of WNTs undoubtedly affects WNT pathways and thus participates in the pathogenesis of diseases. There are 19 members of human WNTs, WNT1, WNT2, WNT2b, WNT3, WNT3a, WNT4, WNT5a, WNT5b, WNT6, WNT7a, WNT7b, WNT8a, WNT8b, WNT9a, WNT9b, WNT10a, WNT10b, WNT11 and WNT16. The expression levels of WNTs, binding receptors, and activated WNT pathways are diverse in different tissue types, which endows the complexity of WNT pathways and multifarious biological effects. Although abundant studies have reported the role of WNTs in the pathogenesis of NSCLC, it still needs further study as therapeutic targets for lung cancer. This review will systematically summarize current research on human WNTs in NSCLC, from molecular pathogenesis to potential clinical practice.

## Introduction

The latest statistics on global cancer data show that lung cancer has become the most common type of cancer with the highest morbidity and leading cause of death worldwide and also in China, among which the incidence and mortality of men rank first, and those of women rank third and second, respectively [1]. According to the histopathological characteristics, lung cancer is divided into non-small cell lung cancer (NSCLC) and small cell lung cancer (SCLC). NSCLC accounts for about 85% of the total incidence of lung cancer and mainly includes three types: adenocarcinoma, squamous cell carcinoma, and large cell carcinoma [2]. Lung adenocarcinoma (LUAD) and lung squamous cell carcinoma (LUSC) account for 60–70% of all lung cancers. Although surgery is regarded as the first choice of treatment for lung cancer, about 70% of patients have progressed to metastasis at diagnosis, or relapse after initial surgery or radiotherapy [3]. At present, the 5-year survival rate of NSCLC patients is as low as 15%, the time from diagnosis to death for most patients in the advanced stage is less than 18 months, and the therapeutic effects of surgery, radiotherapy, and chemotherapy are poor [4, 5].

Over the past decade, immunotherapy and targeted therapy have made substantial progress and significantly prolonged the progression-free survival (PFS) of patients with NSCLC [6, 7]. However, immune checkpoint inhibitors (ICIs) and targeted therapies also bring about some immune-related adverse events [12–14]and other unexpected adverse reactions such as thrombocytopenia, hypertension, and hyponatremia [8–10]. Therefore, it is still necessary to strengthen the research on these drugs to prevent these adverse reactions and to elucidate some other underlying molecular pathogenesis of NSCLC. The pathogenesis of NSCLC is very intricate, involving the abnormal transduction of many signaling pathways, including WNT, tyrosine kinase, Notch, EGFR, Hedgehog, etc. EGFR, which promotes malignant proliferation, metastasis, and angiogenesis of cancer cells by activating intracellular RAS/RAF/MEK/MAPK, PI3K/PTEN/AKT and STAT3 signaling pathways, and inhibits apoptosis of cancer cells, is expressed in over 60% of lung cancer and regarded as an important target for prognosis evaluation and treatment of NSCLC [11]. Abnormal activation of the Hedgehog pathway also enhances the stemness of cancer stem cells (CSCs) and the proliferation of cancer-associated fibroblasts (CAFs) [12]. In addition, the occurrence, metastasis and radiotherapy tolerance of NSCLC are also related to the over-activation of Notch pathway, and abnormal increase in the expression of Notch1 and Notch3 is detected in about 30–40% of NSCLC cases [13, 14]. As conserve pathways that determine the embryonic development and tissue homeostasis in multicellular organisms, WNT signaling pathways play important role in regulating the expression of genes involved in multiple cellular processes, including cell differentiation, proliferation, migration and apoptosis. Therefore, its dysregulation undoubtedly results in disease etiology like tumorigenesis of NSCLC [15, 16].

Although several elaborated review articles have systematically discussed the role of WNT signaling pathways in lung physiology and their dysregulation in the process of lung pathology such as NSCLC lesions, little is known regarding the role of different WNT ligands (WNTs) in the occurrence and progression of NSCLC [17–20]. Here, we will summarize the current insights into the WNTs in NSCLC, from molecular pathogenesis to clinical practice.

## The mechanisms of signal transduction of WNT pathways

### WNT ligands

WNT family contains many homologous genes that are highly conserved during evolution. The WNT gene is named after the wingless (wg) of Drosophila and the int-1 gene of mice [21]. Mutations in the wg gene in Drosophila could produce morphological defects, and the insertion of the mammary tumor virus gene also activates the int-1 gene in mice and promotes tumor formation [22]. Subsequently, a variety of WNT homologous genes were found in most organisms, from nematodes to humans. Among them, Drosophila and mice have 4 and at least 18 WNT genes, respectively [23]. At present, 19 WNT genes have been found in humans, which encode WNT1, WNT2, WNT2b, WNT3, WNT3a, WNT4, WNT5a, WNT5b, WNT6, WNT7a, WNT7b, WNT8a, WNT8b, WNT9a, WNT9b, WNT10a, WNT10b, WNT11 and WNT16 proteins [24]. These cysteine-rich secretory proteins act on different cells and perform a series of functions through paracrine or autocrine [25, 26]. It is widely believed that the aberrant expression of WNT ligands and mediated dysregulation of different WNT signaling pathways exert very important role in the occurrence and progression of most human malignancies, including cancers of the nervous system, the digestive system, the respiratory system, the urogenital system and the musculoskeletal system [17, 27–32].

### The signal transduction of the canonical WNT pathway

According to different downstream signaling cascades, WNT signaling pathways are divided into β-catenin-dependent canonical WNT pathway and β-catenin-independent noncanonical WNT/Ca^2+^ and WNT/planar cell polarity (PCP) pathways, [33, 34] among which the mechanism in the signal transduction of canonical WNT pathway is much clearer. Specifically, in the absence of WNTs, β-catenin is phosphorylated by the destruction complex composed of GSK3β, CK1α, AXIN and APC in the cytoplasm, and then degraded by E3 ligase SCF^βTrCP^-mediated ubiquitination. CK1α phosphorylates β-catenin at serine 45 (S45) and GSK3β phosphorylates β-catenin at S33, S37 and threonine 41. AXIN and APC are responsible for the coupling of β-catenin to GSK3β. Therefore, the truncated mutation of APC could disrupt its binding ability to AXIN and affect the recruitment of β-catenin by the destruction complex. When the secreted WNTs are sufficient, they will bind to different frizzled (FZD) receptors and co-receptors LRP5/6, phosphorylate the intracellular proline-serine-rich regions in LRP6, and then recruit and inhibit GSK3β. DVL is also recruited to bind to the intracellular domain of FZD via its DEP domain to form oligomers, followed by AXIN and GSK3β, where AXIN will bind to the DIX domain of DVL. Therefore, the dissociation of AXIN from the destruction complex and binding to DVL determines the switch of the canonical WNT pathway from an inactive state to an active state. Elevated AXIN levels inactivate the WNT pathway, while elevated DVL levels exert an opposite effect. Because of the dissociation of the destruction complex, β-catenin is accumulated in the cytoplasm and translocated into the nucleus to bind to DNA-binding proteins such as TCF/LEF transcription factors, relieve the inhibitory effect of Groucho transcription inhibitors on TCF/LEF, and recruit transcription co-activators CBP and p300 to activate TCF/LEF, thus regulating the transcription of downstream target genes that determine cell proliferation, differentiation and apoptosis [35, 36]. Therefore, the abnormal signal transduction of the canonical WNT pathway will activate the transcription of a variety of tumor-related target genes, such as c-Myc, Cyclin D1, and vascular endothelial growth factor (VEGF), thereby inducing the malignant transformation of normal cells or promoting the malignant proliferation and metastasis of cancer cells and tumor angiogenesis [37–39]. In addition, the canonical WNT pathway also interacts with TGF-β and Notch pathways to promote tumorigenesis and epithelial-mesenchymal transition (EMT) processes [40]. Interestingly, activation of certain noncanonical WNT pathway, such as WNT5a/Ca^2+^ pathway, can even inhibit the canonical WNT pathway [41]. In conclusion, canonical WNT pathway is an important pathway that determines the occurrence, progression, and metastasis of cancers. Therefore, it is of great significance to further clarify its molecular mechanism for the diagnosis and treatment of malignancies [35, 42].

## The role and action mechanisms of WNTs in tumorigenesis and progression of NSCLC

**Fig. 1:**
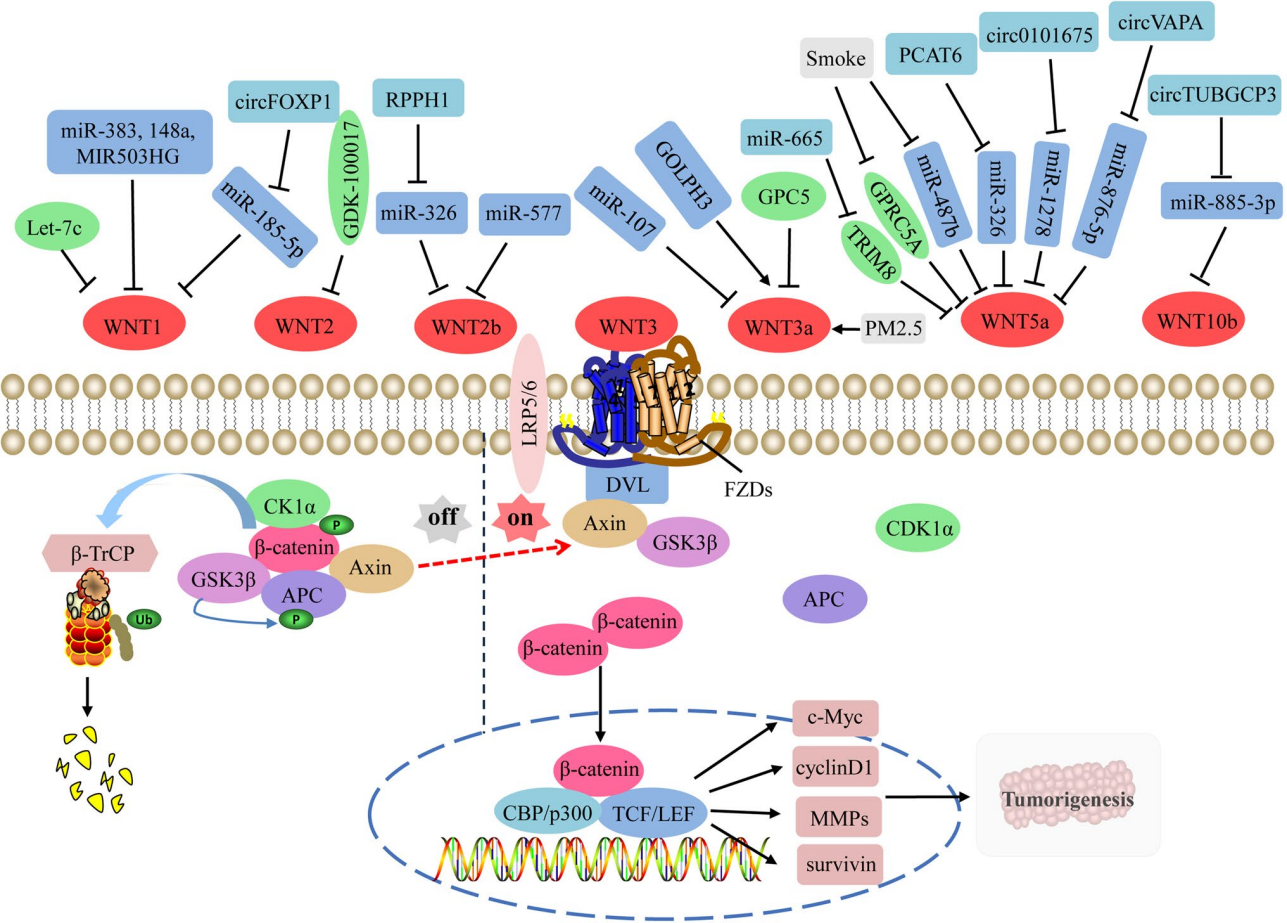
Regulatory functions of oncogenic WNTs and related upstream regulators on the canonical WNT pathway in NSCLC. In the absence of WNTs, the cytoplasmic β-catenin is phosphorylated by CK1α and GSK3β in destruction complex, followed by ubiquitination by its cognate E3 ligase, SCF^βTrCP^. When the secreted WNTs are sufficient, they will bind to different FZD receptors (FZDs) and co-receptors LRP5/6, phosphorylate LRP6 and then recruit DVL to the intracellular domain of FZD to form oligomers, then, AXIN and GSK3β dissociate from the destruction complex and bind to DVL. Therefore, β-catenin can be accumulated in the cytoplasm and then translocated into the nucleus to bind to DNA-binding proteins such as TCF/LEF transcription factors, and recruit transcription co-activators CBP and p300 to activate TCF/LEF, thus regulating the transcription of downstream tumor-related target genes that determine cellular proliferation, differentiation and apoptosis, such as c-Myc, cyclinD1, MMPs and survivin. During the onset and progression of NSCLC, Let-7c, miR-383, miR-148a, lncRNA-MIR-503HG, miR-185-5p, miR-326, miR-577, miR-107, miR-326, miR-1278, miR-876-5p, miR-885-3p, cicFOXP1, GPC5, GPRC5A and TRIM8 could block the signaling transduction of the canonical WNT pathway by inhibiting the activities of WNTs or down-regulating their expression, whereas circFOXP1, lncRNA RPPH1, GOLPH3, smoke, PM2.5, lncRNA-PCAT6, circ101675, circVAPA and circTUBGCP3 could activate the canonical WNT pathway by over-activating WNTs or up-regulating their expression. *APC* adenomatosis polyposis coli, *β-TRCP* β-transducin repeat-containing protein, *CBP* CREB-binding protein, *CK1α* casein kinase 1α, *DVL* disheveled, *FOXP1*, forkhead box protein P1, *FZDs* frizzleds, *GDK-100017* 2,3,6-trisubstituted quinoxaline derivative, *GOLPH3* Golgi phosphoprotein 3, *GPC5* glypican-5, *GPRC5A* G protein coupled receptor family C group 5 type A, *GSK3β* glycogen synthase kinase 3β, *LEF* lymphoid enhancer-binding factor, *MMPs* matrix metalloproteinases, *NSCLC* non-small cell lung cancer, *PCAT6* prostate cancer-associated transcript 6, *RPPH1* ribonuclease P RNA component H1, *TCF* T-cell factor, *TRIM8* tripartite motif-containing 8, *TUBGCP3* gamma tubulin complex component 3

**Fig. 2:**
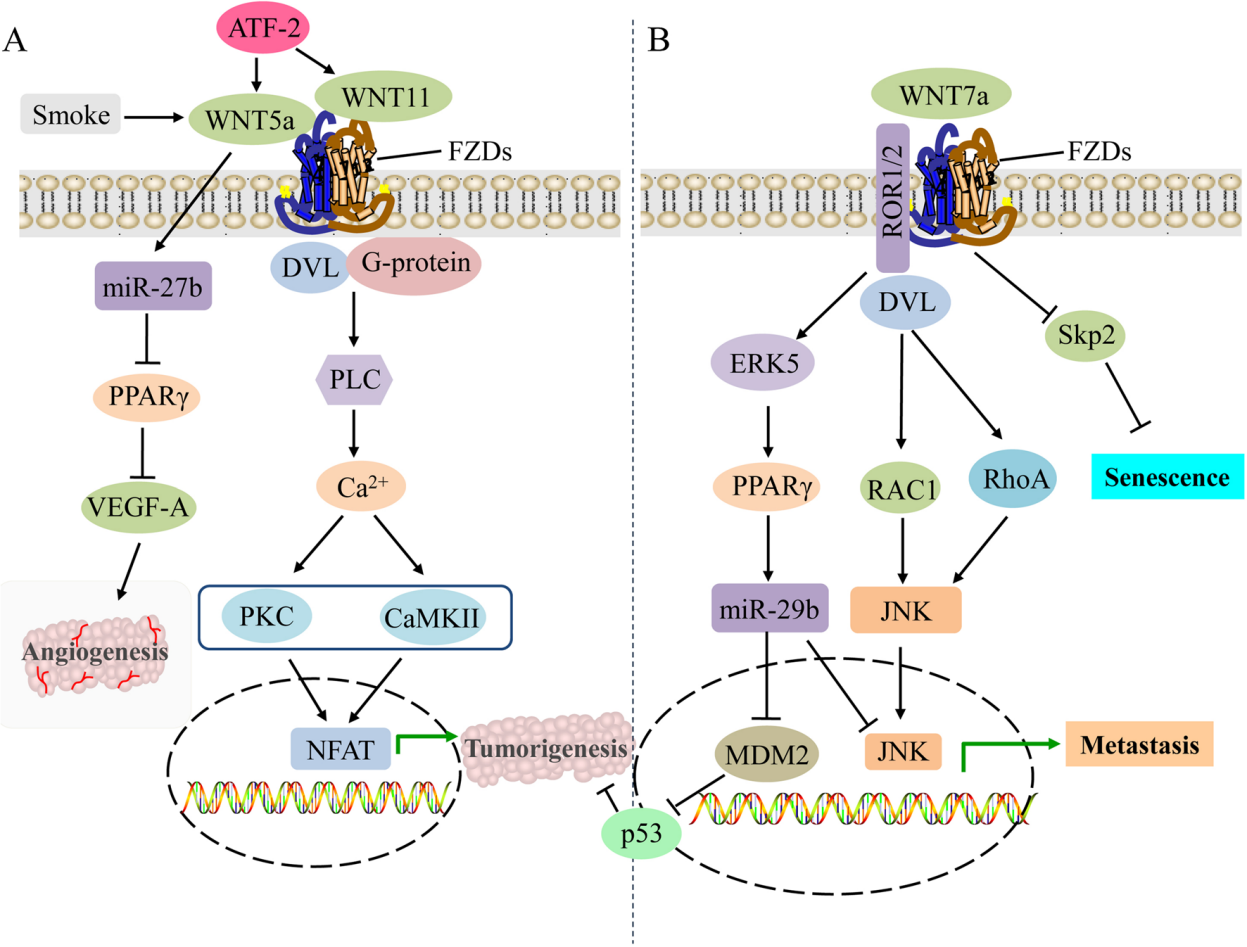
Regulatory functions of anti-cancer WNTs by activating the noncanonical WNT pathways in NSCLC. **A** WNT5a and WNT11 bind to FZDs and G protein subunit to recruit DVL, which triggers PLC activity and promotes intracellular Ca^2+^ release, and further activates calcium-dependent PKC and CAMKII signaling pathways, thus to regulate the expression of downstream genes to promote the proliferation and invasion of NSCLC cells by inducing the accumulation of nuclear NFAT transcription factors. WNT5a also inhibits VEGF-A-induced endothelial cell migration and motility to promote angiogenesis by inducing miR-27b and direct consequence of PPARγ reduction in NSCLC cells. Smoke and ATF-2 could activate the noncanonical WNT pathway by up-regulating the expression of WNT5a and WNT11. **B** The β-catenin-independent Wnt/PCP/JNK pathway is initiated by the cumulative binding of WNT7a to the ROR1/2-FZDs complex, then DVL is activated to bind to some small Rho GTPases such as RAC1 and RhoA to trigger JNK. This results in the inhibition of transformed cell growth but enhancement of migration and invasion of NSCLC cells. The binding of WNT7a to FZD9 inhibits cellular transformation and proliferation of NSCLC cells by activating the tumor suppressor PPARγ via the ERK5-dependent pathway, thus to relieve the inhibitory effect of MDM2 on p53 tumor suppressor pathway by inducing the expression of anti-cancer miR-29b, and promotes epithelial differentiation through activating JNK pathway and the resultant upregulation of cadherins. Wnt7a also triggers the cellular senescence of lung cancer via the inactivation of Skp2, a key negative regulator of cellular senescence. *CAMKII* calmodulin-dependent protein kinase II, *DVL* disheveled, *ERK5* extracellular signal-regulated kinase 5, *JNK* JUN N-terminal kinase, *MDM2* murine double minute 2, *NFAT* nuclear factor of activated T cells, *NSCLC* non-small cell lung cancer, *PCP* planar cell polarity, *PKC* protein kinase C, *PLC* phospholipase C, *PPARγ* peroxisome proliferator–activated receptor-γ, *RAC1* Rac family small GTPase 1, *ROR1/2* receptor tyrosine kinase-like orphan receptor 1/2, *Skp2* S-phase kinase-associated protein-2, *VEGF-A* vascular endothelial growth factor-A

The expression levels, the binding receptors, and activated WNT pathways are different due to the large number of WNT family members. However, there is growing evidence that the aberrant expression of WNTs is closely related to the occurrence and progression of NSCLC, which may serve as important indicators for the early diagnosis and prognosis evaluation of NSCLC [26].In the following sections, we specifically focus on the changes and action mechanisms of different WNTs (Table 1) in the pathogenesis of NSCLC (Figs. 1 and 2), thus providing novel biomarkers and drug targets for the diagnosis and treatment of NSCLC.

**Table 1:** Oncogenic and anti-cancer human WNT ligands (WNTs) in the pathogenesis of NSCLC by regulating the canonical and noncanonical WNT pathways

WNT Ligands	Expression level	Effect	Type of WNT pathway
WNT1	Increased [45, 47, 52]	Oncogenic	Activating canonical [45, 46, 52]
WNT2	Increased [55, 59]	Oncogenic	Activating canonical [60, 61]
WNT2B	Increased [64]	Oncogenic	Activating canonical [64, 71]
WNT3	Increased [77]	Oncogenic	Activating canonical [78]
WNT3A	Increased [81]	Oncogenic	Activating canonical [81, 82, 161]
WNT4	Decreased [131]	No data in NSCLC	
WNT5A	Decreased [107]	Anti-cancer	Inhibiting canonical [107]
	Increased [92, 95]	Oncogenic	Activating canonical [92, 95, 96, 101],
			Activating noncanonical [105, 106]
WNT5B	Increased	Oncogenic	No data in NSCLC
WNT6	Increased [131]	No data in NSCLC	
WNT7A	Decreased [113, 120, 121]	Anti-cancer	Activating canonical [113] Activating noncanonical [114, 115, 117, 122]
WNT7B	Increased [131]	Oncogenic [132]	No data in NSCLC
WNT8A	No data in NSCLC		
WNT8B	Increased [131]	No data in NSCLC	
WNT9A/WNT9B	Decreased [131]	No data in NSCLC	
WNT10A	Increased [131]	No data in NSCLC	
WNT10B	Increased [131]	Oncogenic [133]	No data in NSCLC
WNT11	Increased [127]	Anti-cancer	Inhibiting canonical [127]
			Activating noncanonical [105, 127]
WNT16	Increased [131]	No data in NSCLC	

### WNT1

WNT1 belongs to the proto-oncogene family and is highly conserved in evolution. Its expression level was increased in lung cancer, prostate cancer, and cervical cancer, [43, 44] elevated WNT1 expression was also detected in over 1/3 of NSCLC cases, and was positively correlated with the expression levels of β-catenin, Cyclin D1, and c-Myc. In addition, regardless of the TNM stage of NSCLC, the increased expression levels of WNT1 and β-catenin foreshadowed poor prognosis of NSCLC patients after surgery, [45] suggesting that the increased expression level of WNT1 is an important mechanism leading to the over-activation of canonical WNT pathway in NSCLC. WNT1 not only accelerates the proliferation of tumors by upregulating c-Myc, but also promotes the tumor malignant proliferation and angiogenesis by inducing the expression of target genes such as Cyclin D1, VEGF-A, and matrix metalloproteinase 7 (MMP7) [45, 46]. Survivin is an important inhibitor of apoptosis protein and a target gene of the canonical WNT pathway. Interestingly, its expression level was significantly positively correlated with WNT1 in NSCLC tissues, and its elevated expression level was also an important indicator of poor prognosis [47]. Therefore, WNT1 could be used as a potential drug target to treat NSCLC. Previous studies have reported that some noncoding RNAs, such as miR-383, miR-148a and MIR503HG, could inhibit the migration and invasion of NSCLC cells by targeting the expression of WNT1, induce apoptosis and reduce the tumorigenicity of cancer cells in vivo [48-50]. On the contrary, circFOXP1 could relieve the inhibitory effect of miR-185-5p on WNT1 and promote the progression of LUAD [51]. Recently, some studies even found that WNT1 contributes to the pathogenesis of lung cancer by regulating the tumor immune microenvironment. The expression level of WNT1 was inversely correlated with T cell abundance in LUAD tissues. LUAD cells could inhibit the expression of CC/CXC chemokine in intratumoral conventional dendritic cells (cDCs) by using paracrine WNT1 signaling and induce T cell cytotoxicity and immune resistance. Therefore, silencing WNT1 may be a valuable immunotherapeutic strategy to prevent the progression of LUAD [52]. In addition, elevated plasmic WNT1 protein was correlated with the poor prognosis of advanced NSCLC patients during the treatment of ICIs, which can be attributed to the activation of the canonical WNT pathway mediated by WNT1 [53]. WNT1 is also a target of Let-7c and inhibited by it through increased methylation, high Let-7c could therefore suppress EMT and further potentiate the osimertinib action on NSCLC cells with EGFR T790M mutations [54]. In conclusion, WNT1 promotes the pathogenesis of lung cancer, and inhibition of its overexpression by appropriate methods has a potential therapeutic effect on this disease.

### WNT2

As an important member of the WNT family, WNT2 mainly functions as an oncogene [55]. Over-activation of the canonical WNT pathway mediated by WNT2 was found in many types of cancers, including the colorectal cancer, gastric cancer, breast cancer, and lung cancer [56–58]. Studies have found that the expression level of WNT2 protein in NSCLC tissues and serum of LUAD patients was significantly increased, and was correlated with poor outcomes of patients, it is therefore an important indicator for the diagnosis and prognosis evaluation of NSCLC [55, 59]. In NSCLC cell lines, silencing WNT2 by siRNA or antagonizing WNT2 by anti-WNT2 monoclonal antibody could reduce the cytoplasmic β-catenin level and TCF/LEF transcriptional activity, and induce cellular apoptosis. Similarly, overexpression of construct dominant negative WNT2 reduced the tumorigenicity of NSCLC cells in vitro and in vivo via inhibiting FZD8-mediated activation of WNT2/β-catenin pathway [60]. Moreover, a small molecule inhibitor 2,3,6-trisubstituted quinoxaline derivative, GDK-100,017, was found to inhibit cell proliferation of WNT2 overexpressing NSCLC cells and enhance their sensitivity to radiotherapy in a dose-dependent manner by targeting the canonical WNT pathway [61]. Therefore, targeted silencing of WNT2 expression may be a new therapeutic approach to treat NSCLC.

### WNT2b

As a paralogue of WNT2, WNT2b also promotes the progression and metastasis of malignancies such as head and neck squamous cell carcinoma, malignant pleural mesothelioma, ovarian cancer, and pancreatic cancer, enhances chemotherapy resistance, and leads to poor prognosis by activating the canonical WNT pathway [62-68]. Both mRNA and protein expression levels of WNT2b were elevated in NSCLC cell lines, and overexpression of WNT2b promoted the proliferation, colony formation, and EMT process of NSCLC cells [64]. Interestingly, WNT2b and WNT5a are highly expressed in NSCLC cells and stromal cells and may induce the polarization of tumor-associated macrophages (TAMs) to M2 status to strengthen the tumor progression [69]. On the contrary, miR-577 inhibited the proliferation and EMT of NSCLC cells by interfering with the expression of WNT2b and related canonical WNT pathway, while lncRNA RPPH1 relieved the inhibitory effect of miR-326 on WNT2b expression, and enhanced the invasion ability, EMT and CDDP resistance of NSCLC cells [64, 70]. Therefore, inhibition of WNT2b expression has a potential therapeutic effect on NSCLC, for example, the adenoviral vector carrying shRNA against WNT2b not only induced the apoptosis of several Wnt2b-overexpressing human tumor cells by downregulating c-Myc and survivin, but also exerted a strong antitumor activity in the intrapleural lung cancer model of Wnt2b-overexpressing lung cancer xenografts [71, 72].

### WNT3

As an important member of the WNT family, WNT3 promotes the occurrence and progression of many malignancies, including liver cancer, gastric cancer, and colorectal cancer, by activating the canonical WNT pathway [73-76]. Compared with that in normal tissues, the expression of WNT3 in lung cancer tissues was significantly increased and positively correlated with the expression levels of c-Myc, survivin, and Ki-67. In addition, the expression level of WNT3 in LUSC was higher than that in LUAD, and a higher WNT3 level predicted stronger invasiveness of NSCLC [77]. On the contrary, knockdown of WNT3 in NSCLC cells suppressed cellular proliferation, invasion and metastasis, and induced apoptosis by inhibiting the canonical WNT pathway [78]. Therefore, WNT3 may be another target to treat NSCLC.

### WNT3a

WNT3a is highly homologous to WNT3, but with a 15% difference in the amino acid sequences. WNT3a is highly expressed in gastric cancer, colorectal cancer, prostate cancer, and breast cancer, and could enhances the development and metastasis of these malignancies by activating ERK and canonical WNT pathways [75, 79]. Moreover, WNT3a was found to increase the metastatic abilities of NSCLC cells by promoting the expression of Notch3, N-cadherin and vimentin, and cause EMT morphological changes and F-actin reorganization [80]. Recently, Song et al. found Golgi phosphoprotein 3 (GOLPH3) was overexpressed in NSCLC tissues, and promoted the secretion of exosomal WNT3a and activation of canonical WNT pathway via increasing exosome-localized cytoskeleton-associated protein 4, thus enhancing the metastasis and CSC-like phenotype in NSCLC [81]. Interestingly, fine particulate matter (PM2.5) also increases the risk of lung cancer by promoting WNT3a levels in secreted exosomes and subsequent activation of the canonical WNT pathway. However, these exosomes only enhanced the proliferation of NSCLC cells and exhibited no effect on their invasion and migration [82]. Glypican-5 (GPC5) is a member of heparin sulfate proteoglycan and exerts as a metastasis suppressor in LUAD. Wang et al. found that the expression levels of GPC5 and WNT3a were negatively correlated in LUAD, and GPC5 could suppress the proliferation and metastasis of LUAD cells by inactivating the canonical WNT pathway by competitively binding to WNT3a [83]. In addition, miR-107 is another negative regulator of WNT3a, which inhibits the invasion and EMT of NSCLC cells by suppressing WNT3a and FGF7 expression [84]. In conclusion, these findings provide a theoretical basis for using WNT3a as a drug target to treat lung cancer.

### WNT5a

WNT5a is a para- and autocrine β-catenin-independent ligand that has been shown to inhibit or induce cancer [85, 86]. The expression level of WNT5a is elevated in some cancers and exerts as an oncogene, such as breast cancer, pancreatic cancer, prostate cancer, and gastric cancer [87-89]. In contrast, WNT5a inhibits the progression of breast cancer and liver cancer [90, 91]. Therefore, its expression level and function depend on specific cancer types and tumor microenvironments. The expression level of WNT5a was increased in over 60% of NSCLC cases, especially in lung tumors obtained from smokers and male patients. Moreover, its expression level was higher in LUSC tissues than that in LUAD counterparts [92]. Previous studies have shown that cigarette smoke induced lung carcinogenesis by activating the noncanonical WNT5a/PKC signaling and AKT, or by relieving the inhibitory effect of miR-487b on WNT5a expression and mediated activation of canonical WNT pathway [93, 94]. The expression of WNT5a was positively correlated with the expression of β-catenin, VE-cadherin, MMP2, MMP9 and VEGF-A in NSCLC [92, 95]. Overexpression of WNT5a could promote the colony formation, migration, invasion, EMT and metastasis by activating the canonical WNT pathway [92, 96]. In addition, WNT5a in NSCLC cells could increase the expression of β-catenin and VEGF-A in stromal cells through tumor-stroma interaction, thus promoting tumor angiogenesis [95]. Consistently, high level of WNT5a also inhibits VEGF-A induced angiogenesis in NSCLC squamous cells by inducing miR-27b and the direct consequence of PPARγ reduction [97]. Therefore, NSCLC patients with higher WNT5a expression levels had advanced TNM stages and poor outcomes [95, 98]. On the contrary, silencing WNT5a expression inhibited the malignant phenotype of NSCLC [96, 99]. Recently, miR-1278 and miR-876-5p were found to inhibit the progression of NSCLC by directly downregulating the expression of WNT5a, whereas circ0101675 and circVAPA acted as competing endogenous RNA to relieve the inhibition of miR-1278 and miR-876-5p on WNT5a separately [100, 101]. Similarly, miR-665 activated the WNT5a/β-catenin pathway by inhibiting the expression of TRIM8, enhancing the malignant progression of LUSC [102]. In contrast, sevoflurane, a volatile anesthetic frequently used in surgery, could inhibit the proliferation and invasion, and induce cancer cell apoptosis of LUAD and SCLC cells by blocking the lncRNA PCAT6/miR-326/WNT5a/β-catenin pathway [103]. GPRC5A is a lung tumor suppressor and is often expressed at a low level in smoking lung cancer patients. A recent study has found that cigarette smoke extract could inhibit the expression of GPRC5A in normal human lung epithelial cells and lung cancer cells, inducing the expression of WNT5a and the pathogenesis of lung cancer [104]. However, WNT5a is not always functioning through the activation of the canonical WNT pathway. Zhang et al. found that ATF-2 enhanced the proliferation and invasion of NSCLC cells by activating WNT5a/Ca^2+^ pathway [105]. In addition, the activation of WNT5a/PKC pathway could enhance the stemness of NSCLC cells and inhibit cell apoptosis and their sensitivity to chemotherapies by increasing endoplasmic reticulum release of Ca^2+^, PKC, and CaMKII and the subsequent activation of NF-kB signaling. This effect was strikingly reversed by PKC inhibitor GF109203X, [106] which also provides new insights for further understanding the role of noncanonical WNT pathways in lung carcinogenesis and chemoresistance. Reference: Kindly check whether the inserted [Page range] for references [31, 53, 69, 81, 86, 96, 124, 127, 148, 166] are appropriate.Patients with advanced NSCLC are prone to have brain metastasis and poor prognosis, and may be enhanced by EGFR mutation. However, a recent study reported that WNT5a protein was significantly decreased in brain metastasis samples and EGFR-mutant tissues because of the activation of its upstream negative regulatory ERK1/2-E2F1 pathway. Overexpression of WNT5a could inhibit the progression of EGFR mutant NSCLC by blocking the canonical WNT pathway, [107] indicating that WNT5a plays an anti-cancer role in the development of such NSCLC. Therefore, the role of WNT5 in the pathogenesis of NSCLC needs to be studied further.

### WNT5b

WNT5b is highly homologous to WNT5a, but 18% difference in the amino acid sequences. WNT5b could activate the canonical and noncanonical WNT pathways, and may play an opposite role in different types of cancers [108]. Elevated expression of WNT5b promoted the progression of oral squamous cell carcinoma and breast cancer but predicted a better prognosis for glioma patients [108–110]. WNT5b is also highly expressed in LUAD and is positively associated with the TNM stage and poor prognosis. Therefore, overexpressing its negative upstream regulator, miR-5587-3p could suppress the progression of LUAD by interfering cell cycle and modulating amino acid metabolism [111]. Interestingly, WNT5b-associated exosomes secreted from colorectal cancer cells and pancreatic cancer cells could stimulate the migration and proliferation of NSCLC cells in a paracrine manner [112]. However, there is a still lack of study on the effect and action mechanism of WNT5b in the pathogenesis of NSCLC, and needs further study.

### WNT7a

Unlike most WNTs, WNT7a can activate the canonical and noncanonical WNT pathways, but only exerts an anti-cancer effect on lung cancer. The expression of WNT7a is frequently decreased in NSCLC, accompanied by the downregulation of β-catenin and E-cadherin levels [113]. WNT7a not only induces cellular senescence by inactivating S phase kinase-associated protein 2 in a β-catenin-independent manner, [114] but also inhibits the progression of lung cancer by activating E-cadherin expression in a β-catenin-dependent manner [113]. On the contrary, WNT7a-null mice displayed E-cadherin to N-cadherin switch and a decrease in the expression of cell senescence markers and related phenotype, suggesting the increased lung tumorigenesis of these mice [114]. Strikingly, several studies performed by Winn and colleagues have found that direct binding of WNT7a to WNT receptor FZD9 in NSCLC cells could inhibit cellular transformation and proliferation by increasing the activity of a tumor suppressor PPARγ in a Gα16/ERK5 pathway-dependent manner and resultant disinhibitory effect of MDM2 on p53 tumor suppressor pathway by inducing the expression of anti-cancer miR-29b, and promote epithelial differentiation of these cells through activating JNK pathway and the resultant upregulation of cadherins [115–118]. Interestingly, *WNT7a* gene is located at the chromosome 3p25 region, which is known as a predilection site of homozygous deletion of many anti-cancer genes [119]. In addition, there is a high percentage of methylation in the promoter region of *WNT7a* gene in NSCLC tissues, which is positively correlated with the advanced TNM stage and may be related to the increased expression of DNA methyltransferase 1 induced by cigarette smoke condensate [120, 121]. However, a recent study reported that WNT7a exerted an antiproliferative effect on NSCLC cells by activating the noncanonical JNK pathway, but enhanced their migration and invasion abilities [122]. In conclusion, WNT7a is an important molecule that inhibits the occurrence and progression of NSCLC, and restoring its expression could be a valuable therapeutic strategy for treating NSCLC.

### WNT11

WNT11 is most homologous to WNT4, but still with 59% difference in the amino acid sequences. Most studies have shown that WNT11 promoted the development of multiple malignancies, such as breast cancer, colon cancer, and prostate cancer, by activating the canonical and noncanonical WNT pathways [123–125]. However, WNT11 was found to inhibit the proliferation and metastasis of liver cancer cells via activating the noncanonical PKC pathway but suppressing the canonical WNT pathway [126]. Although WNT11 is upregulated in LUSC tissues, it inhibits cellular adhesion of NSCLC cells via suppressing the expression of E-cadherin and canonical WNT pathway, but activating nonclassical WNT pathway [127]. WNT11 is also lowly expressed in chemo-resistant SCLC cells, indicating its upregulation may contribute to the treatment of lung cancer [128]. Recently, Ito and colleagues found that the increased acetylation level of H3K27, an enhancer of the *WNT11* gene caused by the upregulation of ASCL1 and its recruitment to oncogenic SOX2 may be an initiating factor in the cause of WNT11 upregulation and resultant progression of lung cancer [129, 130]. In addition, ATF-2 is found to enhance the proliferation and invasion of NSCLC cells by inducing the expression of WNT11 and mediated activation of WNT11/Ca^2+^ pathway [105]. In short, WNT11 is mainly carcinogenic in the development of lung cancer, and inhibiting its overexpression may aid the treatment of this disease.

###  The other WNTs

The latest bioinformatics analysis on NSCLC datasets showed that the expressions of WNT4, WNT9a, and WNT9b were decreased in LUAD tissues, whereas expressions of WNT6, WNT7b, WNT10a, WNT10b and WNT16 were increased in LUAD and LUSC tissues [131]. GATA4 is an important tumor suppressor in lung cancer and could induce the senescence of lung cancer cells via upregulating several miRNAs that target TGFB2 mRNA and ensuing downregulation of WNT7b expression, suggesting WNT7b may play a carcinogenic role in lung cancer [132]. WNT10b exerts a similar role in this pathogenesis because circTUBGCP3 promotes the progression of LUAD by competitively binding miR-885-3p to relieve its inhibition on WNT10b/β-catenin pathway [133]. Unfortunately, the role and action mechanism of WNT4, WNT6, WNT8a, WNT8b, WNT9a, WNT9b, WNT10a, and WNT16 in the pathogenesis of NSCLC has not been reported and needs more studies.

## The application of WNT-based drugs in the treatment of NSCLC

### Overall profile

In recent years, cancer therapies targeting WNT pathways have attracted more attention with the deeper understanding of the signal transduction mechanisms of WNT pathways, and the development of multiple targeted drugs is ongoing. The pharmacological mechanisms of these drugs specifically include the following aspects: inhibiting the secretion of WNTs and their binding to receptors; reducing the expression of β-catenin or inactivating its activity; blocking the signaling transduction of canonical WNT pathway, restoring the function or expression of certain negative regulators in this pathway, and improving the sensitivity of resistant cancer cells to anti-NSCLS drugs (Table 2).

**Table 2:** Selected classes of existing/potential WNT-based drugs in the treatment of NSCLC.

Agents	Classes	Targets	Pharmacological Mechanisms	Stage of Development	References
LGK974	Specific small molecules	↓PORCN	PORCN inhibitor; inhibits the progression of NSCLC in vitro and in vivo; prolongs the survival time of mice with advanced LUAD	Phase 1	[134, 135]
ETC-159					
CGX1321					
RXC004					
XAV939		↓TNKS	Stabilizes AXIN to promote the degradation of β-catenin; inhibits the proliferation and migration of NSCLC cells; improves the effect of cisplatin; enhances the sensitivity of EGFR-mutated NSCLC cells to EGFR-TKIs through the combination of XAV939 and EGFR-TKIs	Discovery	[138, 139, 162]
AZ1366					
GDK100017		↓WNT2	Inhibits the canonical WNT signaling; suppresses the proliferation of NSCLC cells; enhances the sensitivity of NSCLC cells to radiotherapy	Discovery	[61, 138, 142]
ICG-001		↓β-catenin/CBP	Reduces the transcriptional activation effect of β-catenin/TCF; inhibits the proliferation and invasion of NSCLC cells by abolishing the upregulating effect of BCAT1	Phase 1	[143]
Procaine	Anesthetic drug	↑WIF-1	Reactivates WIF-1 from a previously silenced Methylation in NSCLC cells; downregulates the WNT canonical pathway; induces the autophagy and apoptosis of NSCLC cells	Discovery	[148]
Procainamide	Arrhythmic drug				
OMP-18R5	Monoclonal antibody	↓FZD7	Competitors of WNTs; blocks the activation of the canonical WNT pathway; inhibits the growth of NSCLC cells in vitro and in vivo	Phase 1	[136, 137, 163]
OMP-54F28	Decoy Receptor	↓Multiple WNTs			
Curcumin	Plant-based agents	↓MAT1/WNT/β-catenin	Inhibits the proliferation and invasion of NSCLC cells; induces G0/G1 phase arrest	Phase 1	[144, 164]
25-OH-PPD		↓β-catenin/ Cyclin D1/ c-Myc	Inhibits the growth of xenograft tumors in mice; decreases the expression levels of β-catenin and its downstream targets Cyclin D1, CDK4, and c-Myc in lung cancer cells	Discovery	[145]
BDMC		↑WIF-1	Inhibits TGF-β1-induced EMT in highly metastatic lung cancer cells		[149]
Trifluoperazine	Antipsychotic agent	↓canonical WNT signaling	Inhibits the drug resistance of lung CSCs	Discovery	[151, 159, 160]
HY1-Pt	CK2 inhibitor				
Garcinol	Plant-based agent				

*↑/↓* increase/decrease expression level or activity, *BDMC* bisdemethoxycurcumin, *CBP* CREB-binding protein, *CK2* casein kinase 2, *EGFR-TKIs* epidermal growth factor receptor-tyrosine kinase inhibitors, *EMT* epithelial-mesenchymal transition, *FZD* frizzled, *LUAD* lung adenocarcinoma, *MAT1* metastasis-associated protein 1, *NSCLC* non-small cell lung cancer, *PORCN* porcupine, *TCF* T-cell factor, *TGF-β1* transforming growth factor beta 1, *TNKS* tankyrases, *WIF-1* WNT inhibitory factor 1, *25-OH-PPD* 25-hydroxyprotopanaxadiol

### Inhibiting the activity of WNTs

The normal secretion of WNTs and binding to receptors depend on their lipidation with palmitic acid at two conserved serine residues by an acyltransferase named porcupine (PORCN) at the post-translational level, and most WNTs are upregulated in lung cancer tissues. Therefore, inhibiting the expression or activity of Porcupine could theoretically reduce the over-activation of WNT pathways. It has been reported that silencing Porcupine expression reduced the expression of β-catenin in NSCLC cells [24]. However, Porcupine is indispensable for the maintenance of the physiological WNT pathways, excessive inhibition in it may have potentially toxic effects on normal tissues. As a specific inhibitor of Porcupine, LGK974 significantly inhibited the progression of NSCLC in vitro and in vivo, and prolonged the survival time of mice with advanced LUAD. Strikingly, the formation of cyclodextrin: LGK974 inclusion complexes could enhance the solubility and bioavailability of LGK974 in mice and reduce its intestinal toxicity [134]. Unfortunately, none of Porcupine-related inhibitor is commercially available, and LGK974 (NCT01351103), ETC-159 (NCT02521844), CGX1321 (NCT02675946), and RXC004 (NCT03447470) are still tested in phase I clinical trials [135].

### Competitive binding to WNT receptors

The competitive binding of WNT receptors is another effective strategy to inhibit the canonical WNT pathway. OMP-18R5 is a monoclonal antibody targeting FZD7, which can competitively bind to five FZDs due to the high homology of FZD family members. Therefore, this antibody could inhibit the growth of NSCLC cells in vitro and in vivo by blocking the activation of the canonical WNT pathway induced by WNTs [136]. Similarly, the fusion protein OMP-54F28 could bind to the cysteine-rich domain of FZD8 with its Fc domain to antagonize WNT signaling, suppressing the growth of multiple cancers and tumorigenicity of CSCs [137].

### Promoting the degradation of β-catenin

Currently, a variety of small molecule inhibitors and natural compounds targeting β-catenin have been developed, given its essential role in the canonical WNT pathway. Tankyrases are important regulators in the canonical WNT pathway, XAV939 specifically binds to them to stabilize AXIN in the destruction complex, thus promoting the phosphorylation and subsequent degradation of β-catenin. Interestingly, XAV939 has been reported to inhibit the proliferation and migration of NSCLC cells by targeting the canonical WNT pathway. Moreover, the combination use of XAV939 and cisplatin improved the therapeutic effect of cisplatin and reduced its adverse reactions [138]. The activating mutation of EGFR exists in over 60% of NSCLC cases and is recognized as an important target for cancer therapy. It has been found that the combination of tyrosine kinase inhibitors (EGFR-TKIs) and XAV939 or another tankyrase inhibitor AZ1366, could significantly inhibit canonical WNT pathway and EGFR protein phosphorylation, enhancing the sensitivity of EGFR-mutated drug resistant NSCLC cells to EGFR-TKI [139, 140]. Dong and colleagues found that 2,3,6-trisubstituted quinoxaline derivative (GDK100017), a novel small molecule inhibitor of the canonical pathway, could suppress the proliferation of NSCLC cells and enhance their sensitivity to radiotherapy by blocking WNT2-induced activation of canonical WNT pathway [61, 141, 142]. In addition, some small molecules also inhibit the canonical WNT pathway in other ways. ICG-001 is a selective inhibitor of the canonical WNT pathway, which competes against β-catenin to bind to the N-terminal region of CBP and reduces the transcriptional activation effect of β-catenin/TCF. Recently, ICG-001 was found to inhibit the proliferation and invasion of NSCLC cells in vitro by abolishing the upregulating effect of BCAT1 on the canonical WNT pathway [143]. Curcumin is a naturally occurring phenolic compound and found to inhibit the proliferation and invasion of NSCLC cells through metastasis-associated protein 1 (MTA1)-mediated inactivation of canonical WNT pathway [144]. 25-hydroxyprotopanaxadiol is a natural compound isolated from Panax ginseng, and its derivatives exert anti-cancer activities by inhibiting the canonical WNT pathway in NSCLC cells [145].

### Activating the expression of negative regulators

Contrary to the upregulation of WNT activators, negative regulators in canonical WNT pathway are often downregulated in many malignancies. WIF-1 is a member of secreted FZD-related protein family and could suppress the activation of the canonical and noncanonical WNT pathways by competitively binding to WNTs. Some studies have found that both the transcription and translation levels of the *WIF-1* gene were reduced in NSCLC tissues because of the hypermethylation in its promoter region [146, 147]. Interestingly, the anesthetic procaine and the antiarrhythmic procainamide, inhibitors of DNA methylation, were found to restore the expression of WIF-1 and ensuing inactivation of canonical WNT pathway in NSCLC cells, suggesting these two drugs have potential therapeutic effects on lung cancer [148]. Similarly, bisdemethoxycurcumin inhibited TGF-β1-induced EMT in highly metastatic lung cancer cells by upregulating expression of WIF-1 protein [149]. In addition, WIF-1 was found to induce the autophagy and apoptosis of NSCLC cells by inhibiting DVL2-mediated activation of canonical WNT and PI3K/AKT/mTOR pathways [150]. In summary, upregulating the expression of negative regulators in canonical WNT pathway is also a potential approach for treating lung cancer.

### Inhibiting the drug resistance of NSCLC cells

Certain WNTs, such as WNT2b, WNT6 and WNT11, are more lowly expressed in the chemo-resistant lung cancer cells, and cumulative evidence suggests that the activation of canonical WNT pathways, partially attributed to the upregulation of some key oncoproteins, such as serine-arginine protein kinase 1 (SRPK1), ras-associated binding protein 25 (Rab25) and B cell lymphoma 9 (BCL9), contributes to the resistance of NSCLC cells to anti-cancer drugs such as cisplatin and the first-line EGFR-TKIs through inducing the expression of several genes involved in multi-drug resistance such as anti-apoptotic isoform of Bcl-xL, Oct4 and Nanog, and genes involved in the proliferation and maintenance of CSCs like CD44 and CD133 [151-156]. Therefore, the combination of inhibitors of canonical WNT pathway and current anti-NSCLC drugs would undoubtedly overcome all manner of resistance and improve the therapeutic effectiveness. For example, trifluoperazine, an antipsychotic agent, enhances the inhibitory of EGFR-TKIs and overcomes drug resistance in lung CSCs by blocking the canonical WNT pathway [151]. Moreover, Cai et al. showed antagonism of miR-128-3p could reverse the chemoresistance of highly malignant NSCLC cells by inhibiting canonical WNT signaling-induced CSC-like properties [157]. Recently, Yan et al. found that inhibiting the activity of DCLK1, a CSC marker, restored the sensitivity of EGFR-TKIs-resistant NSCLS cells through suppression of canonical WNT pathway and cancer stemness [158]. In addition, Wang et al. demonstrated a novel CK2 inhibitor, HY1-Pt, could reverse cisplatin-induced resistance by suppressing CSCs through canonical WNT pathway [159]. Interestingly, some natural compound such as garcinol was also found to inhibit CSC-like phenotypes via inactivation of canonical WNT pathway and STAT3 in NSCLC [160]. Generally, negative regulators of the canonical WNT pathway have a synergistic effect with anti-NSCLS drugs and are promising drug candidates in controlling drug resistant NSCLC cells.

Currently, the research on targeted therapies for lung cancer based on WNT pathways is in its infancy due to the complexity of these pathways. It still needs to deeply understand the mechanisms of disorders of WNT pathways in the pathogenesis of lung cancer and drug resistance, thus screening out reliable biomarkers for early clinical diagnosis and prognosis estimation of NSCLC and therapeutic targets for this disease. Moreover, it is necessary to strengthen the research on the WNT pathways in cancer immunotherapy.

## Conclusion

WNTs are a class of secretory proteins that play important roles in embryonic development and tissue homeostasis in a paracrine or autocrine manner. However, the abnormal signal transduction of WNT pathways induced by the ectopic expression or dysfunction of WNTs is one culprit for the occurrence and progression of many human malignancies, including NSCLC. In this review, the changes and action mechanisms of human WNTs in the pathogenesis of NSCLC and related therapeutic strategies were discussed separately. Except for WNT5a, WNT7a, and WNT11, most WNTs exert a carcinogenic role by regulating the canonical and/or noncanonical pathways. Additionally, the role of some WNTs has never been elucidated in NSCLC. In summary, we hope this review will be helpful to gain a deeper understanding of the role of WNTs and mediated WNT pathways in the pathogenesis of NSCLC and arouse more researchers to develop WNTs-based therapeutic approaches for NSCLC.
